# Changes in pregnancy-related hormones, neuromechanical adaptations and clinical pain status throughout pregnancy: A prospective cohort study

**DOI:** 10.1371/journal.pone.0314158

**Published:** 2025-02-21

**Authors:** Catherine Daneau, François Nougarou, Jacques Abboud, Stephanie-May Ruchat, Martin Descarreaux

**Affiliations:** 1 Department of Anatomy, Université du Québec à Trois-Rivières, Québec, Canada; 2 Department of Electrical and Computer Engineering, Université du Québec à Trois-Rivières, Québec, Canada; 3 Department of Human Kinetics, Université du Québec à Trois-Rivières, Québec, Canada; The University of British Columbia, CANADA

## Abstract

During pregnancy, increased hormonal levels contribute to ligament laxity of the pelvis and could predispose to lumbopelvic pain. The main objective of this study was to assess changes in pregnancy-related hormones, neuromechanical adaptations and clinical pain status throughout pregnancy. An exploratory objective was to examine the possible association between those variables. Twenty-eight pregnant women participated in the study. At each trimester, they provided a blood sample (to measure relaxin, estrogen and progesterone), completed questionnaires assessing clinical status (functional disability, risk of poor prognosis of prolonged lumbar disability, avoidance behaviors, anxiety and pain catastrophizing), and were asked to perform a flexion-relaxation task (erector spinae electromyography and trunk kinematics). Results showed that throughout pregnancy, nocturnal and diurnal lumbopelvic pain intensity and related-disability, risk of poor lumbopelvic pain prognosis as well as avoidance behaviors increased, while pain catastrophizing decreased. Neuromechanical characteristics of flexion-relaxation task, including low back muscle activity and trunk kinematics, were similar across the three trimesters. Positive correlations were found between disability and estrogen levels (changes between first and second trimester, p = 0.05), and estrogen and diurnal lumbopelvic pain intensity (change between second and third trimester, p = 0.02). A positive correlation was also found between weight and the Pelvic Girdle Questionnaire score (changes between second and third trimester, p = 0.05). Negative correlations were found between weight (change between first and second trimester) and lumbopelvic maximal angle (p = 0.003), FRP onset for pelvic (p = 0.04) and lumbopelvic (p = 0.003) angles as well as FRP cessation for lumbopelvic angle (p = 0.001). These results show that, in pregnant women, pain and disability are associated with hormonal changes rather than trunk neuromechanical characteristics during a flexion-relaxation task. These results suggest that the flexion-relaxation task may not be an appropriate proxy to study vertebral and pelvic muscle control in pregnant women.

## Introduction

Pregnancy is characterized by multiple adaptations including anatomical, biomechanical, and physiological changes over a relatively short period of time. These changes are challenging the women’s body and might lead to pain development and associated disability in the lumbar and pelvic region. Pain felt in both regions is called lumbopelvic pain (LBPP) [1]. A recent observational pilot study, which aimed to determine the prevalence of low back pain (LBP), pelvic girdle pain (PGP) and LBPP among 287 pregnant women showed that the prevalence of those three “lower back” conditions increases throughout pregnancy [2]. Two studies, by the same researcher, showed a prevalence of 18% in the first trimester for pregnant women with LBPP ([3]) and a prevalence of 33% in the third trimester for women with LBP and/or PGP ([4]).

Physiological changes during pregnancy include the fluctuation of different hormones such as relaxin, estrogen, progesterone, prolactin, oxytocin, growth hormones and parathyroid hormone levels. These hormones all have a specific impact on the woman’s body. An experimental study conducted in guinea pigs showed that hormonal administration of relaxin or a combination of relaxin and estrogen showed an increase in tibial displacement (12.8% and 13.6% respectively), compared to pre-hormonal administration [5]. During pregnancy, relaxin levels peak in the first trimester whereas estrogen and progesterone reach their peak levels at the end of the pregnancy [6], and also have a similar impact on ligamentous laxity [7]. These ligament changes are believed to yield increased instability in the lumbar and pelvic region [8]. During the first trimester, pregnant women had similar metacarpophalangeal and lumbopelvic joint ligament laxity as compared to women who were not pregnant. Metacarpophalangeal joint laxity then increased by 11% between the first and second trimesters and remained afterwards stable until delivery, while laxity of the lumbopelvic region significantly increased in the third trimester. Additionally, the Beighton score was significantly higher in the second trimester of pregnancy compared to other trimesters.

Women are also exposed to biomechanical adaptations throughout pregnancy, such as change in magnitude and distribution of loads (fetal loads) [9], sustained strain of pelvic structures and muscle weaknesses [10] as well as muscular imbalance and joint misalignments [11], which could modify their lumbar configuration and pelvic position [12]. Lumbopelvic stability in pregnant women is ensured by a complex system including passive components (bones, ligaments, tendons, cartilages), active structures (muscles) and the nervous system which provides the sensory, motor, and central integration and process components involved in maintaining functional joint stability [13,14]. In the neutral position, the stability of the lumbopelvic girdle is mainly ensured by trunk muscles, whereas the contribution of both the active and passive structures will provide the required stability of the lumbar and pelvic regions during flexion, extension, rotation and torsion of the trunk. The passive structures are the main structures providing stability to the lumbopelvic joint as it approaches a full range of motion [13]. Although most studies investigating lumbopelvic stability have not been conducted in pregnant women, the increased motion of the pelvic joints and pubic symphysis seems to play a role in the pathogenesis of LBPP [15]. The flexion-relaxation phenomenon (FRP) has been identified in the literature as a method to evaluate the complex interaction between the different components of spinal stability. The FRP is defined as a silencing of myoelectric activity of the lumbar erector spinae muscles during full-trunk flexion [16]. It is considered a robust and reproducible neuromuscular response, which has been identified in most healthy individual, including pregnant women [17]. This neuromuscular response is likely triggered by increased mechanical loads in the ligaments and discs of the lumbar spine [18]. The amplitude of electromyography (EMG) responses and kinematics characterizing FRP can be influenced by many factors including loading of the trunk [19], lumbopelvic posture [19,20], angular trunk velocity [21], task repetition [22] and muscular fatigue [23,24]. Most importantly, persistent activation of the lumbar erector spinae muscles during a full trunk flexion is typically observed in individuals with LBP, a protective “splinting” response to increase lumbar stabilization in response to pain or tissue injury [25–27]. Thus, because of its high ecological value and its well-documented reproducibility [28], the FRP is considered to be an interesting experimental model to assess the various processes underlying trunk muscle adaptation and changes in stability during pregnancy.

Only a few studies have investigated the effect of pregnancy on FRP biomechanical parameters. In the third trimester, healthy pregnant women show a decreased trunk flexion and increased erector spinae EMG amplitude during trunk flexion movement, compared to women who were never pregnant [29]. A study conducted by Sihvonen et al. (1998) evaluated the intensity of LBP and disability in the second and third trimester of pregnancy in pregnant women who either had or did not have LBP prior to their pregnancy [17]. Results from this study indicated a significant positive correlation between the intensity of current pain and the level of erector spinae muscle activity during trunk flexion movement. It also reported that back muscle activity during bending measured in the 1^st^ trimester of pregnancy was significantly and positively correlated with pain intensity measured in the 3^rd^ trimester [17]. The study, however, did not explore the association between hormonal, neuromechanical and clinical changes.

The main objective of this study was to assess changes in levels of pregnancy-related hormones, trunk neuromechanical characteristics, and clinical LBPP status throughout pregnancy. An exploratory objective was to examine the possible association between those variables. It was hypothesized that neuromechanical changes would occur during pregnancy and that these changes would be correlated to changes in pregnancy-related hormones and clinical pain outcomes through the different trimesters.

## Materials and methods

### Participants

Using *a priori* sample size calculation, it was determined that 25 pregnant women would be required to detect a significant difference between trimesters in muscle activation (10% change from one trimester to another) (β = 0.8 and p < 0.05). For this prospective cohort study, 28 pregnant women, whose characteristics are presented in Table 1, were recruited between July 23, 2019 and March 11, 2021 at the local gynecology and obstetrics clinic, which provides prenatal care to approximately 75% of pregnant women in the region. Social media (Facebook) was also used to recruit participants. Since we were interested in LBPP development and evolution, pregnant women with and without LBPP were eligible.

**Table 1:** Participants’ characteristics.

	Mean ± SD (N = 28)
Age (years)	29.8 ± 3.6
Parity	
0 (n)	42.9% (n = 12)
≥1 (n)	57.1% (n = 16)
Self-reported pre-pregnancy weight (kg)	64.64 ± 17.35
Height (m)	1.64 ± 0.09
Pre-pregnancy BMI (kg/m^2^)	24.00 ± 6.91
Education levels	
No diploma	3.6% (n = 1)
High school diploma	0% (n = 0)
Post-secondary diploma	96.4% (n = 27)

SD: standard deviation, BMI: body mass index.

To be included in the study, women had to be 18 years of age or older, and carrying one fetus. Women presenting any of the following conditions were excluded from the study: inflammatory arthritis of the axial skeleton, collagenosis, advanced osteoporosis, vertebral surgery, neuromuscular disease, malignancy, uncontrolled hypertension, infection or other non-mechanical pain, radiculopathy, progressive neurological deficit, myelopathy, lumbar disc herniation as well as severe and incapacitating pain limiting the ability to fully perform the trunk flexion-relaxation task. This study was approved by the Université du Québec à Trois-Rivières ethics committee (CER-18-252-07.07). All pregnant women who participated in this study provided their written informed consent before the first experimental session.

### Laboratory assessment protocol and hormonal dosage

**Fig 1:**
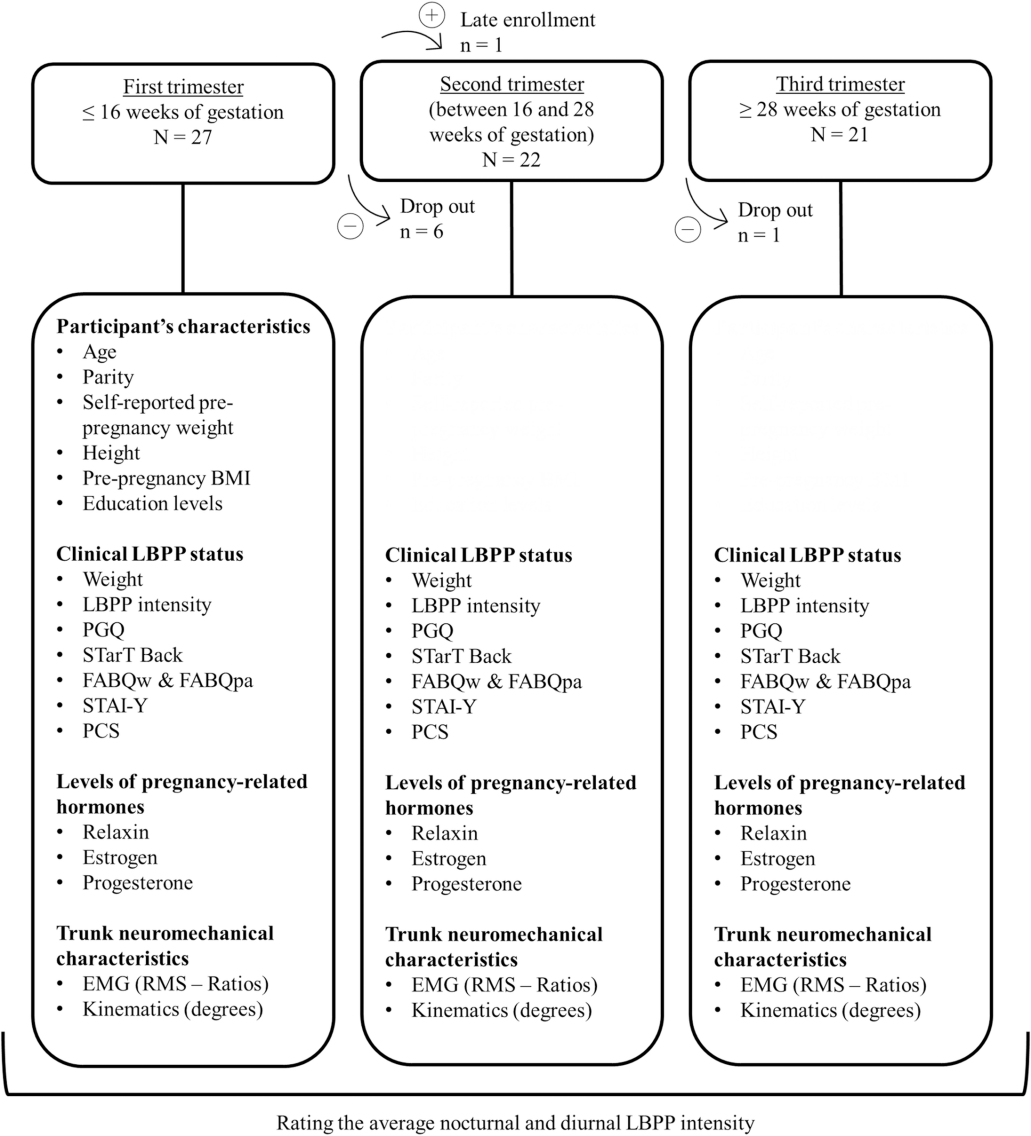
The number of women participating to each assessment. BMI: body mass index; LBPP: lumbopelvic pain; PGQ; Pelvic Girdle Questionnaire; FABQ: Fear Avoidance Beliefs Questionnaire work and physical activity scale; STAI: State-Trait Anxiety Inventory, Y Form; PCS: Pain Catastrophizing Scale; EMG: electromyography; RMS: root mean square.

All women were assessed at each trimester according to the following timeframe: first experimental session (before 16 weeks of gestation), second experimental session (between 16 and 28 weeks of gestation) and third experimental session (after 28 weeks of gestation). Each experimental session was identical (i.e., collected the same information and included the same evaluation) and lasted about 60 minutes. During an experimental session, women had to complete different questionnaires evaluating different clinical parameters related to LBPP. Women were then asked to complete a flexion-relaxation task and a blood sample was taken the same day. Between the first and the last experimental session, weekly average LBPP levels were monitored (see Fig 1).

### Clinical outcomes

Questionnaires assessed the functional disability (Pelvic Girdle Questionnaire), risk of poor prognosis of prolonged lumbar disability (STarT Back Screening Tools), avoidance behaviors related to work and physical activity (Fear Avoidance Beliefs Questionnaire), anxiety (State-Trait Anxiety Inventory, Y Form) and pain catastrophizing (Pain Catastrophizing Scale). Sociodemographic characteristics and anthropometry measurements were collected during the first experimental session. At each evaluation sessions, weight was measured using a scale in the laboratory. Between the first and the last experimental session, women received a weekly text message via their cellphone asking them to rate the average LBPP intensity on a VAS (0 meaning “no pain” and 100 meaning “the worst pain”) for day and night. These scores were used to compute mean LBPP score at each trimester. A score of 18-19 on 100 is considered the minimal clinically important difference for pain VAS [30] while the clinically significant LBPP value on a VAS was 34/100 [31]. This represents pain status with a limited impact on the functioning of individuals suffering from chronic musculoskeletal pain.

### Neuromechanical variables

The flexion-relaxation task was conducted according to the following sequence and instructions:

- Phase 1) initial position (quiet standing, knees in extension, arms crossed on the chest) for 2 seconds,
- Phase 2) trunk flexion completed in 5 seconds,
- Phase 3) full-trunk flexion maintained for 3 seconds and
- Phase 4) trunk extension back to initial standing position completed in 5 seconds (quiet standing, arms crossed on the chest).

The standardization of the task was controlled by an auditory metronome, as well as verbal instructions. The task was performed 5 times, and a 1-minute rest was allowed between each trial.

### Electromyography

Surface EMG (sEMG) was collected using four bipolar electrodes (Model DE2.1, Delsys Inc., Boston, MA, USA) applied on both sides of the spine over the right and left lumbar erector spinae muscles (L1 and L5). For each experimental session, the EMG electrodes were placed by the same assessor. Skin was prepared by gently exfoliating the skin with fine-grade sand paper (Red Dot Trace Prep, 3M; St. Paul, MN, USA) and wiping the skin with alcohol swabs.

At each trimester, and prior to the FRP task, a submaximal voluntary back extension contraction in a control position, visually assessed, was completed (standing with their trunk straight, women were asked to hold an approximative 45-degree trunk flexion for 5 seconds against gravity without adding a supplemental load). EMG data from the submaximal voluntary contraction were used to normalize EMG data during the flexion-relaxation task. When performing the sub-maximal contractions (as well as for all trials of the trunk flexion-extension task), participants were asked to breathe normally throughout the movement in order to avoid the Valsalva maneuver. The use of submaximal contractions for participants unable to perform maximal contractions such as pregnant woman or individuals with pain remains an interesting alternative for subsequently standardizing the recorded data [32].

### Kinematics

Kinematics data were collected using the Optotrak Certus motion analysis system (3 motion capture devices: Northern Digital, Waterloo, Ontario, Canada). Seven kinematics markers were placed by the same assessor on anatomical landmarks on the right side of the participant. The locations of the markers were 1) the lateral malleolus, 2) the lateral condyle of the femur, 3) the greater trochanter, 4) the anterior superior iliac spine (ASIS), 5) S2, 6) L1 and 7) T12. The following angles were created using the kinematics markers: lumbar, pelvic and lumbopelvic angles.

### Data analysis

The EMG activity was recorded using the Bagnoli-8 EMG acquisition system (common mode rejection ratio of 115 dB at 60 Hz, input impedance of 1MΩ) and sampled at 1000 Hz with a 12-bit analog to digital converter (PCI-6024E; National Instruments, Austin, Texas, USA). To remove artifacts related to electrodes movement and the power line, a 20–400 Hz band pass 4^th^ order Butterworth filter and notch filters at 60 Hz and its harmonics were applied to all EMG signals. Kinematic data were sampled at 100 Hz and overall signal was rebased to zero to allow comparison between women.

**Fig 2:**
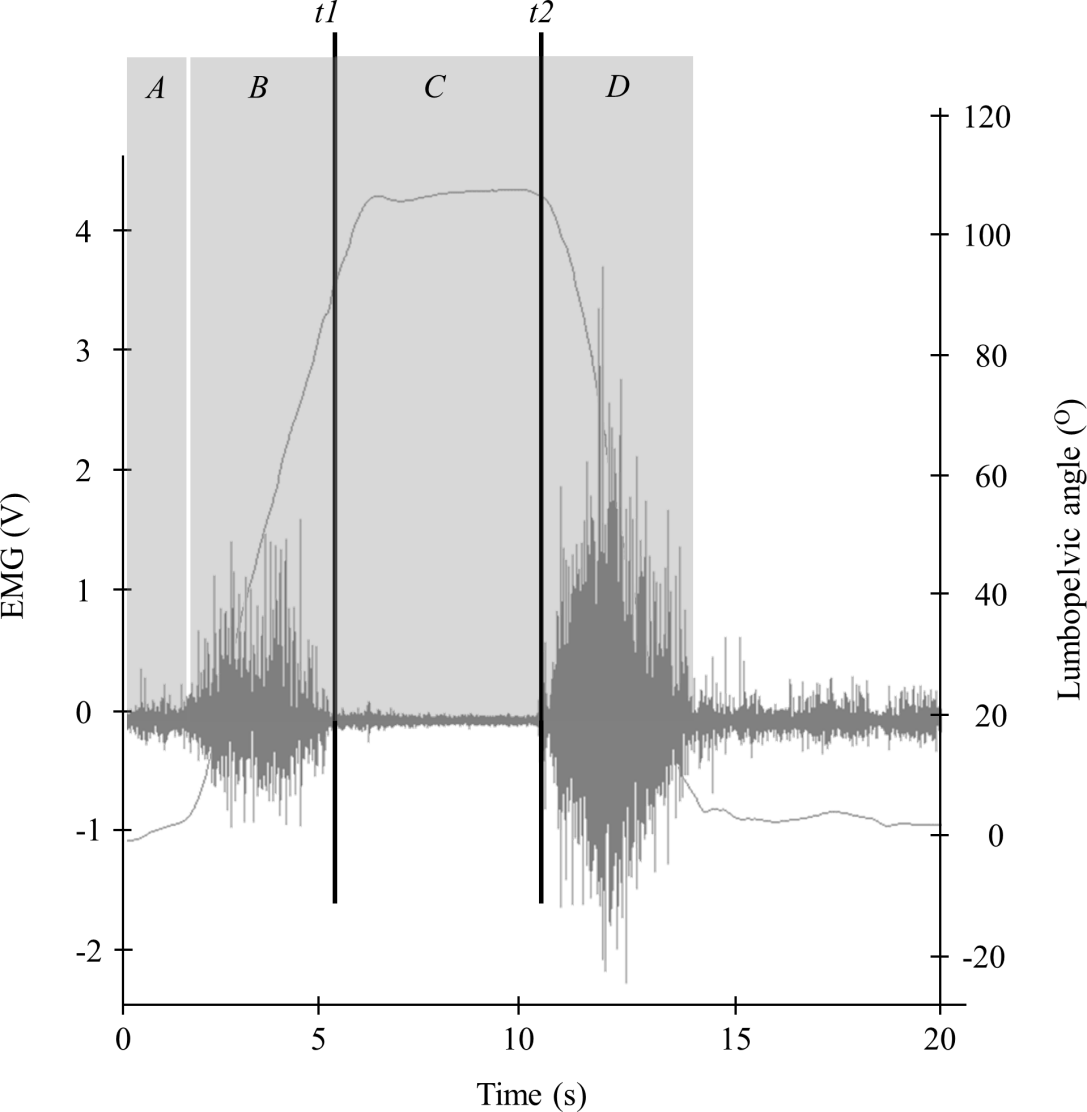
FRP time events on raw EMG signals. A: initial position, B: trunk flexion, C: full-trunk flexion maintained, D: trunk extension back to initial standing position.

The visual marking of the FRP time events was performed on the rectified EMG signals superposed to kinematics data: the first mark at the EMG onset of the full-trunk flexion phase (named *t*_1_) and the second mark at the EMG cessation of the full-trunk extension phase (named *t*_2_). Then, 4 time periods were used on the EMG signals to characterize the FRP: baseline RMS during phase 1 (between 0.5s and 2s), a first burst of muscle activity RMS during phase 2 (lumbar erector spinae eccentric contraction), EMG silence RMS observed during the full trunk flexion (phase 3, between *t*_1_ and *t*_2_) and a second burst of muscle activity RMS during phase 4 (lumbar erector spinae concentric contraction) (Fig 2). A root mean square (RMS) value was computed for each of the 4 phases. Normalized RMS EMG values were obtained by dividing the mean RMS during each phase by the RMS value obtained during the submaximal voluntary contraction.

The flexion-relaxation ratio (FRR) was obtained by dividing the RMS value of the trunk flexion phase by the RMS value of the full-trunk flexion. All data were analyzed on Matlab (Matlab Release 2022b, The MathWorks, Inc.; Massachusetts, United States).

For kinematics data, the three angles of interest were lumbar, pelvic and the combination of lumbar and pelvic (lumbopelvic) angles. The lumbar angle was formed from the vector (L1 – S2) and the vector (ASIS – S2). The pelvic angle resulted from the (ASIS – S2) and (greater trochanter – lateral condyle of the femur) vectors. Finally, the lumbopelvic angle was formed from the addition of the lumbar and pelvic angles. Total lumbar, pelvic and lumbopelvic angles were obtained and respectively corresponding to EMG onset of FRP during the trunk flexion phase and to EMG cessation of FRP during the trunk extension phase. Relative onset and cessation lumbopelvic angle were also calculated using the absolute onset and cessation angles divided by the maximal lumbopelvic angle reached during full flexion. All parameters from the 5-trunk flexion-extension trials were averaged and subsequently used for statistical analyses.

### Hormonal variables

A blood sample was taken for hormonal dosage (relaxin, estrogen and progesterone) done by a private laboratory. All samples were stored at −20^o^C until the analysis were done, and no sample was diluted. Levels of relaxin were estimated manually using the quantitative enzyme-linked immunosorbent assay (ELISA). According to the laboratory who did this test has an intra- and inter-assay coefficients of variance of 2.7% and 7.2% respectively. The sensibility of the assay was 4.57 pg/mL. Levels of estrogens were estimated using the electrochemiluminescence immunoassay (ECLIA) on the Roche platform. This test has an intra- and inter-assay coefficients of variance of 4.1% and 5.5% respectively. The sensibility of the assay was 18.4 pmol/L. Levels of progesterone were estimated using the chemiluminescent macroparticle immunoassay (CMIA) on the Abbott platform. This test has an intra- and inter-assay coefficients of variance of 2.9% and 3.2% respectively. The sensibility of the assay was ≤  0.1 ng/mL.

### Statistical analyses

Normality of all data was verified by visual inspection of data distribution and with the Shapiro-Wilk test. The effect of time (i.e., trimesters of pregnancy) on levels of pregnancy-related hormones, trunk neuromechanical characteristics (EMG and kinematics) and clinical LBPP status (pain scores and disability) were assessed using repeated measures ANOVA and its equivalent for nonparametric data (Friedman ANOVA). When necessary, the Tukey post-hoc test (parametric tests) and Wilcoxon Matched Pairs Test (nonparametric tests) was performed for pairwise comparisons. Pearson correlation analyses were conducted to assess the linear association between changes (from first to second trimester and from second to third trimester) in the levels of pregnancy-related hormones, trunk neuromechanical characteristics and clinical LBPP status (pain and disability) as well as weight. For all analyses, statistical significance was set at p <  0.05. All statistical analyses were performed with Statistica version 13.5.0.17 (Tibco Statistica 13.5).

## Results

Among the 28 participants included in the study, 20 completed all assessments, 6 completed only the first trimester assessment, 1 completed only the first and second trimester assessments whereas 1 participant completed only the second and third trimester assessments (Fig 1). The participants’ characteristics are presented in Table 1.

### Clinical LBPP status

**Fig 3:**
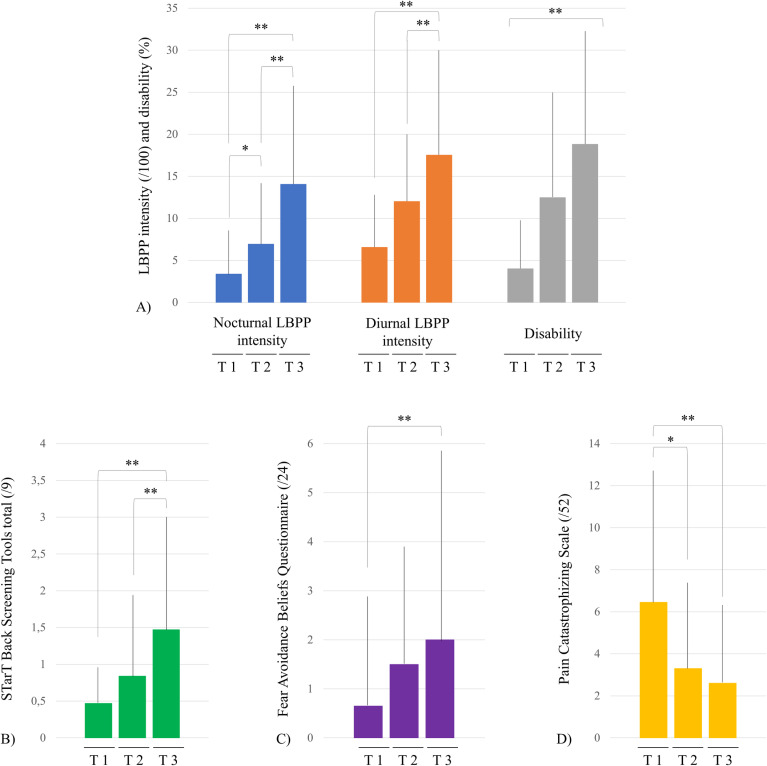
Clinical changes throughout pregnancy. LBPP: lumbopelvic pain; T: trimester. A) Nocturnal LBPP intensity (blue), diurnal LBPP intensity (orange) and disability (grey). B) Risk of poor prognosis of prolonged lumbar disability (STarT Back Screening Tools), C) Avoidance behaviors related to physical activity (Fear Avoidance Beliefs Questionnaire), D) Pain catastrophizing (Pain Catastrophizing Scale). *  **p** < 0.05, ** **p** < 0.001.

Weight significantly increased throughout pregnancy (F(2.38) = 94.35, p < 0.001). Results showed that 70.8% of women reported LBPP (diurnal and/or nocturnal) in the first trimester (17/24), 100% of women in the second trimester (25/25) and 95.7% of women in the third trimester (22/23). From the first to the 3^rd^ trimester, significant increases in nocturnal (χ2 = 24.42, p < 0.001) and diurnal (χ2 = 9.77, p = 0.008) LBPP intensity, as well as in PGQ scores (χ2 = 25.79, p <  0.001), were observed. Similarly, risk of poor prognosis (STarT Back total: χ2 = 13.16, p =  0.001) and fear avoidance beliefs (FABQ physical activity scale: χ2 = 12.17, p = 0.002) significantly increased, and PCS score significantly decreased (χ2 = 9.94, p = 0.007) (Fig 3). All clinical outcomes are presented in Table 2.

**Table 2:** Clinical changes across trimesters of pregnancy.

	Mean ± SE			
	First trimester	Second trimester	Third trimester	*p*
Weight (kg)	70.09 ± 3.02	75.51 ± 2.97	79.45 ± 3.05	< 0.001
	Mean ± SD			
LBPP intensity (/100)				
Nocturnal LBPP	3.37 ± 5.40	6.93 ± 7.11	14.04 ± 12.77	< 0.001^†^
Diurnal LBPP	6.54 ± 6.91	12.01 ± 8.85	17.53 ± 12.54	0.008^†^
Questionnaires				
PGQ^a^	4.00 ± 5.45	12.47 ± 12.94	18.80 ± 14.52	< 0.001^†^
STarT Back^b^	0.47 ± 0.61	0.84 ± 1.07	1.47 ± 1.61	0.001^†^
STarT Back^c^	0.00 ± 0.00	0.11 ± 0.46	0.21 ± 0.54	0.17^†^
FABQw^d^	2.45 ± 3.32	5.25 ± 8.03	4.35 ± 6.28	0.54^†^
FABQpa^e^	0.65 ± 2.30	1.50 ± 2.46	2.00 ± 3.93	0.002^†^
STAI-Y^f^	23.95 ± 3.14	26.10 ± 6.04	25.55 ± 7.37	0.63^†^
STAI-Y^g^	29.65 ± 9.11	31.60 ± 8.76	29.35 ± 8.54	0.24^†^
PCS^h^	6.45 ± 6.28	3.30 ± 4.07	2.60 ± 3.78	0.007^†^

SD: Standard deviation, SE: Standard error,

†Friedman ANOVA. p <  0.05.

^a^PGQ: Pelvic Girdle Questionnaire (% disability),

^b^STarT Back Screening Tools total (/9),

^c^STarT Back Screening Tools subtotal (/5),

^d^Fear Avoidance Beliefs Questionnaire – work scale (/42),

^e^Fear Avoidance Beliefs Questionnaire – physical activity scale (/24),

^f^State-Trait Anxiety Inventory, Y Form – situational anxiety (/80),

^g^State-Trait Anxiety Inventory, Y Form – anxiety traits (/80),

^h^Pain Catastrophizing Scale (/52).

### Levels of pregnancy-related hormones

**Fig 4:**
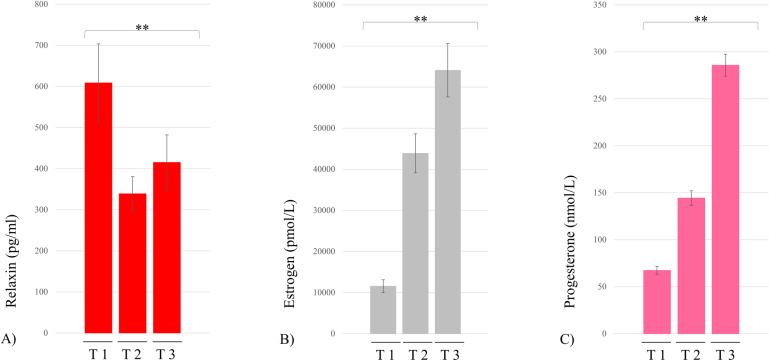
Hormonal changes across the trimesters of pregnancy. T: trimester. A) Levels of relaxin, B) Levels of estrogen (Friedman ANOVA), C) Levels of progesterone. ** **p** ≤ 0.001.

Significant changes in relaxin (F(2.38) = 8.12, p = 0.001), estrogen (χ2 = 34.11, p < 0.001) and progesterone (F(2.36) = 187.09, p < 0.001) levels were observed throughout pregnancy. Tukey post-hoc analyses revealed that relaxin levels at first trimester (Mean_first_: 608.76 ±  94.81) were significantly higher than at the second trimester (Mean_second_: 338.65 ± 42.06, p = 0.001) and at the third trimester (Mean_third_: 415.24 ±  67.01, p = 0.02). Estrogen levels significantly increased throughout pregnancy (Mean_first_: 11,547.11 ± 6695.13; Mean_second_: 43,872.78 ± 20,139.47; Mean_third_: 64,088.39 ± 27,582.36, p < 0.001). Analyses revealed that progesterone levels significantly increased from the first to the third trimester (Mean_first_: 67.34 ± 4.20; Mean_second_: 144.35 ± 7.75; Mean_third_: 285.70 ± 11.68, p < 0.001) (Fig 4).

### Trunk neuromechanical characteristics

The EMG flexion-relaxation ratio did not significantly vary across the three trimesters. Kinematics variables, which include maximal trunk flexion angle, total angles corresponding to EMG onset of FRP (trunk flexion phase) and EMG cessation of FRP (trunk extension phase), as well as the relative onset and cessation lumbopelvic angle, did not significantly change throughout pregnancy (Table 3).

**Table 3:** Neuromechanical changes across the trimesters of pregnancy.

	Mean ± SE			
	First trimester	Second trimester	Third trimester	*p*
EMG (RMS - Ratios)				
EMG L1 left FRR	2.45 ± 0.40	2.68 ± 0.35	2.47 ± 0.39	0.79
EMG L1 right FRR	3.08 ± 0.68	2.89 ± 0.52	3.22 ± 0.62	0.86
EMG L5 left FRR	1.94 ± 0.35	1.94 ± 0.24	2.05 ± 0.43	0.96
EMG L5 right FRR	2.09 ± 2.01	1.90 ± 1.28	2.09 ± 1.49	0.95^†^
Kinematics (degrees)				
FRP maximal flexion angle				
Pelvic maximal angle	47.12 ± 3.24	47.89 ± 3.81	48.81 ± 3.54	0.75
Lumbar maximal angle	17.72 ± 1.69	13.38 ± 2.16	15.17 ± 2.05	0.17
Lumbopelvic maximal angle	66.11 ± 4.57	61.99 ± 5.21	62.73 ± 4.07	0.37
FRP onset (degrees)				
Pelvic angle	42.31 ± 2.91	43.27 ± 3.12	45.44 ± 3.16	0.32
Lumbar angle	18.00 ± 3.71	11,09 ± 2.03	12.07 ± 1.21	0.09
Lumbopelvic angle	58.62 ± 4.41	55.10 ± 4.26	57.41 ± 3.59	0.47
FRP cessation (degrees)				
Pelvic angle	43.47 ± 2.76	45.12 ± 3.94	46.23 ± 4.03	0.56
Lumbar angle	18.50 ± 3.54	11.93 ± 2.16	13.61 ± 1.35	0.12
Lumbopelvic angle	60.89 ± 4.54	58.50 ± 5.39	59.74 ± 4.75	0.77
FRP onset				
Relative lumbopelvic angle	92.20 ± 1.93	91.70 ± 2.05	93.26 ± 1.41	0.53
FRP cessation				
Relative lumbopelvic angle	95.81 ± 1.63	95.69 ± 1.50	96.47 ± 2.44	0.86

SE: Standard error, FRP: flexion-relaxation phenomenon, FRR: flexion-relaxation ratio.

†Friedman ANOVA.

### Exploratory correlations

A significant positive correlation was found between changes in estrogen levels and changes in PGQ score between the first and second trimester (r = 0.46; p = 0.05), indicating that as estrogen levels increased, the level of disability also increased. A significant positive correlation was found between change in estrogen levels and diurnal LBPP intensity from the second to the third trimester (r = 0.53; p = 0.02). Again, as estrogen levels increased, the level of LBPP intensity also increased. A significant positive correlation was found between changes in weight and changes in PGQ score between the second and third trimester (r = 0.44; p = 0.05), indicating that, as weight increased, the level of disability also increased. A significant negative correlation was found between changes in weight and changes in lumbopelvic maximal angle between the second and third trimester (r = −0.69; p = 0.003), indicating that as weight increased, the lumbopelvic maximal angle decreased. Two significant negative correlations were also found between changes in weight and changes in FRP onset, for both pelvic and lumbopelvic angle, between second and third trimester. These results indicated that as weight increased, the FRP onset for pelvic (r = −0.50; p = 0.04) and lumbopelvic (r = −0.69; p = 0.003) angles decreased. Finally, a significant negative correlation was found between changes in weight and changes in FRP cessation (lumbopelvic angle) between the second and third trimester (r = −0.63; p = 0.001), which indicated that as weight increased, the FRP onset (lumbopelvic angle) decreased. All correlations are presented in Tables 4 and 5.

**Table 4:** Correlation between clinical pain and hormonal changes between trimesters.

	Between first and second trimester		Between second and third trimester	
	r	*p*	r	*p*
Relaxin				
Nocturnal LBPP intensity	0.08	0.76	−0.17	0.49
Diurnal LBPP intensity	0.32	0.23	0.04	0.86
PGQ	0.08	0.76	0.07	0.77
Estrogen				
Nocturnal LBPP intensity	0.40	0.11	0.20	0.41
Diurnal LBPP intensity	0.09	0.74	0.53	**0.02**
PGQ	0.46	**0.05**	0.33	0.17
Progesterone				
Nocturnal LBPP intensity	0.13	0.60	−0.42	0.86
Diurnal LBPP intensity	0.23	0.36	−0.14	0.56
PGQ	−0.04	0.88	0.34	0.14

LBPP: lumbopelvic pain; PGQ: Pelvic Girdle Questionnaire.

**Table 5:** Correlation between weight and clinical pain, hormonal changes and neuromechanical changes between trimesters.

	Between first and second trimester		Between second and third trimester	
	r	*p*	r	*p*
Clinical changes				
Nocturnal LBPP intensity	−0.34	0.15	0.17	0.47
Diurnal LBPP intensity	0.04	0.86	−0.03	0.89
PGQ	0.11	0.65	0.44	**0.05**
Hormonal changes				
Relaxin	0.19	0.46	−0.002	0.99
Estrogen	0.30	0.21	0.11	0.66
Progesterone	−0.10	0.69	−0.27	0.25
Neuromechanical changes				
EMG (RMS - Ratios)				
EMG L1 left FRR	−0.05	0.85	−0.32	0.18
EMG L1 right FRR	−0.01	0.97	−0.36	0.13
EMG L5 left FRR	−0.06	0.80	−0.05	0.82
EMG L5 right FRR	−0.16	0.50	0.35	0.13
FRP maximal flexion angle				
Pelvic maximal angle	−0.45	0.07	−0.04	0.87
Lumbar maximal angle	−0.48	0.051	0.16	0.51
Lumbopelvic maximal angle	−0.69	**0.003**	0.09	0.72
FRP onset (degrees)				
Pelvic angle	−0.50	**0.04**	−0.37	0.14
Lumbar angle	−0.44	0.07	0.27	0.28
Lumbopelvic angle	−0.69	**0.003**	−0.04	0.87
FRP cessation (degrees)				
Pelvic angle	−0.35	0.17	−0.26	0.32
Lumbar angle	−0.45	0.06	0.21	0.39
Lumbopelvic angle	−0.63	**0.001**	0.05	−0.83
FRP onset				
Relative lumbopelvic angle	0.04	0.90	−0.35	0.15
FRP cessation				
Relative lumbopelvic angle	−0.11	0.69	−0.11	0.67

FRP: flexion-relaxation phenomenon, FRR: flexion-relaxation ratio, LBPP: lumbopelvic pain; PGQ: Pelvic Girdle Questionnaire.

## Discussion

This study sought to compare the changes in clinical pain status, pregnancy-related hormones and trunk neuromechanical characteristics across the three trimesters of pregnancy. It was also designed to explore the potential correlation between these variables. This study is the first one to explore the evolution of trunk neuromuscular strategies of pregnant women during a flexion-relaxation task.

Results of this study showed that, nocturnal and diurnal LBPP intensity and related disability, risk of poor LBPP prognosis and avoidance behaviors related to physical activity increased throughout pregnancy, while pain catastrophizing decreased. Similar results have been observed in previous studies. Lardon et al. (2018) investigated the prevalence and intensity of pregnancy-related LBPP, anxiety and physical activity levels throughout pregnancy in women who conceived spontaneously or after fertility treatments [33]. Including 59 pregnant women, the study results showed that the prevalence and intensity of LBPP increased during pregnancy, while anxiety decreased from early to mid-pregnancy. Although we did not observe a significant decrease in anxiety in this study, pain catastrophizing significantly decreased throughout pregnancy. Hormonal fluctuations observed in our study are in accordance with fluctuations normally described during pregnancy [6,7]. Neuromechanical characteristics of FRP, including EMG and kinematics, were similar throughout pregnancy. Changes in disability (first to second trimester) was associated with estrogen levels while changes in estrogen and diurnal LBPP intensity were associated during the second and the third trimester respectively. On the other hand, weight was associated with a few clinical and neuromechanical outcome changes occurring during pregnancy including disability and trunk kinematics (maximal angle, FRP onset and cessation). Such results suggest that weight gain in the abdomen and breast may partly explain changes in neuromechanics and perceived physical disability in pregnant women.

### Neuromechanical adaptations and clinical pain status

Sihvonen et al. were the first, in 1998, to describe differences in neuromechanical variables between pregnant women with and without LBPP [17]. Their findings suggested a lack of FRP in pregnant women with LBPP and showed a positive association between pain intensity and trunk muscle activation, which were not observed in the current study since our results failed to identify a correlation between LBPP intensity and lumbar muscle activity. The fact that Sihvonen et al. (1998) assessed pregnant women at 20 and 36 weeks of pregnancy, that the participants had a broader age range and that their LBPP intensity in the 2^nd^ and 3^rd^ trimesters of pregnancy was slightly lower may explain the differences between both studies [17]. A more recent study aimed to analyze movements of the lumbar region and the activation pattern of the lumbar erector spinae muscles in three groups of asymptomatic women: non-pregnant, pregnant and postpartum [29]. The authors reported an increase in muscle activation during the trunk flexion phase for the pregnant group compared to the other two groups. In addition, the onset of FRP flexion appeared later in the trunk flexion phase for pregnant women compared to women in the other two groups. This finding is similar to our study results and suggest that onset and cessation parameters of FRP are partially modulated by increased loading of the spine rather that the presence of pain. Previous studies in healthy adults have also shown the modulatory effect of increased loading on FRP parameters [24,34]. This suggest that pregnant women activate their lumbar muscles for a broader range of motion during the trunk flexion phase, in order to stabilize their lumbar region. Although LBPP during pregnancy may partly arise from changes in neuromuscular stability mechanisms, our results suggest that the FRP may not be an appropriate model to identify trunk neuromechanical adaptations related to pain during pregnancy.

Pain, related disability, and motor control are often considered intrinsically linked; a recent study by Desgagnés et al. (2022) systematically reviewed the evidence regarding the potential differences in lumbopelvic spine motor control between women with pregnancy-related and postpartum LBPP and matched controls [35]. The results derived from the 15 studies included in the review showed quite heterogenous study designs and outcomes (kinematics, EMG or ultrasound). Pregnant women with LBPP (4 studies on kinematic outcomes) showed differences in lumbar spine kinematics during lifting and walking. During post-partum period (two studies on EMG outcomes), women with PGP, compared to pain-free post-partum women, showed higher transversus abdominis activation during leg movements. Given the results of Desgagné’s systematic review, and considering the absence of changes observed in FRP characteristics in the current study and the lack of strong correlation with clinical and hormonal status during pregnancy, one may argue that new tasks, such as walking or stair climbing, or evaluations of LBPP muscle control might be needed to better establish the possible links between LBPP muscle control, hormonal and clinical status.

### Hormonal changes and clinical pain status

A scoping review published in 2021 explored the associations between hormonal changes and clinical pain status in pregnant women with LBPP [36]. This review showed mixed results, with only 4 studies out of 9 suggesting an association between relaxin levels and the presence and intensity of LBPP episodes. One study focused on estrogen and progesterone levels, and found that progesterone levels were higher in pregnant women with LBPP compared to those without LBPP [37]. However, the measurement ranges for hormone levels measurements were rather wide during pregnancy: 6 to 12, 13 to 17, 18 to 22, 24 to 31 and 32 to 38. Estrogen was the only hormone that showed no difference between the two groups of pregnant women, which is inconsistent with our results. Indeed, in the present study, estrogen was the only hormone that was associated with clinical pain status: levels of disability (between the first and second trimester) and diurnal LBPP intensity (between the second and third trimester). Estrogen is believed to increase the elasticity and flexibility of tissues, including those in the pelvic region [7]. The association observed with pain between the second and third trimester could therefore be explained by the gradually increasing level of this hormone during pregnancy. The results of the current study challenge previous findings, and suggest that estrogen may be associated with clinical outcomes of LBPP, while relaxin and progesterone do not seem to be related to clinical LBPP outcomes during pregnancy.

### Hormonal changes and neuromechanical adaptations

Interestingly, our study showed that the relative lumbopelvic FRP onset angles were higher during the trunk flexion phase in pregnant women (93 degrees) compared to previously observed relative angles in non-pregnant individuals (80 degrees) [38]. Although previously described as potential modulating factors involved in LBPP during pregnancy [12], no association was found between hormonal changes and neuromechanical adaptations. In fact, our results suggest that FRP modulation is mostly driven by increased weight during pregnancy rather than hormonal changes and clinical pain status.

### Strength and limitations

One of the strengths of this study is that it focused on three pregnancy hormones that have an impact on ligament laxity in pregnant women, and potentially on the development of LBPP. Most studies identified in the scoping review of Daneau et al. included only relaxin, while only one study explored the association between estrogen, progesterone and LBPP [36]. Another strength is the inclusion of evaluations of hormonal changes, neuromuscular adaptations and clinical pain status of women at each trimester. Additionally, the average nocturnal and diurnal LBPP intensity during each trimester was calculated using weekly data collected via weekly text message assessments. Collecting LBPP intensity data every week may represent a more accurate evolution of LBPP throughout pregnancy.

Limitations of the study include dropouts (21.4%) and partial completion of assessments (7.1%), which in most cases were related to Covid-19 restrictions. Another limitation would be the use of submaximal contractions for EMG data normalization, which can increase variability in muscle activation assessments. However, it is not recommended to perform maximal voluntary contraction in individuals with chronic pain as well as pregnant women [32].The time of day for experimental sessions and blood sample collections were not the same for every women. Since hormones vary between pregnancy weeks, and that women may not be in the same physical condition to perform tests at the beginning and end of the day, future studies should aim at collecting physical and physiological data in the same conditions throughout the trimesters.

The intensity of LBPP in our study was much lower than what has been previously described in the literature (scores between 50 to 60 mm during pregnancy; 20 mm in the first to 75 mm in the third trimester) [39] mostly due to the inclusion of women with LBP history (an important risk factor for LBPP during pregnancy) that were pain free at the first trimester [40]. However, our results may not fully apply to a population of pregnant women with moderate to severe pain and significant functional disability. The onset and evolution of LBPP in women during pregnancy is potentially influenced by several complex interactions between various risk factors, contextual factors and physiological changes and/or neuromechanical adaptations. Some of the changes and adaptations that occur during pregnancy may not necessarily be associated with LBPP, which would explain why motor adaptations may occur before the onset of LBPP.

## Conclusion

In conclusion, this study showed an increase in LBPP intensity and related disability, and in the risk of poor LBPP prognosis throughout pregnancy, along with an increase in avoidance behaviors related to physical activity. However, pain catastrophizing decreased over time. Hormone levels varied throughout pregnancy, in accordance with fluctuations normally described. Neuromechanical characteristics, including EMG and kinematics, remained similar across the three trimesters. Finally, our exploratory analyses showed no correlation between relaxin and clinical pain status but did show correlation between estrogen and LBPP-related disability and diurnal LBPP intensity, challenging previous findings. It also showed some correlation between weight and clinical and neuromechanical changes. More studies are needed to validate the correlations obtained in this study. The study also highlights the need for new functional evaluations of LBPP muscle control in order to better establish the possible links between LBPP muscle control, hormonal and clinical pain status.
